# The Danish High-Risk and Resilience Study—VIA 15 – A Study Protocol for the Third Clinical Assessment of a Cohort of 522 Children Born to Parents Diagnosed With Schizophrenia or Bipolar Disorder and Population-Based Controls

**DOI:** 10.3389/fpsyt.2022.809807

**Published:** 2022-04-04

**Authors:** Anne Amalie Elgaard Thorup, Nicoline Hemager, Vibeke Fuglsang Bliksted, Aja Neergaard Greve, Jessica Ohland, Martin Wilms, Sinnika Birkehøj Rohd, Merete Birk, Anette Faurskov Bundgaard, Andreas Færgemand Laursen, Oskar Hougaard Jefsen, Nanna Lawaetz Steffensen, Anna Krogh Andreassen, Lotte Veddum, Christina Bruun Knudsen, Mette Enevoldsen, Marie Nymand, Julie Marie Brandt, Anne Søndergaard, Line Carmichael, Maja Gregersen, Mette Falkenberg Krantz, Birgitte Klee Burton, Martin Dietz, Ron Nudel, Line Korsgaard Johnsen, Kit Melissa Larsen, David Meder, Oliver James Hulme, William Frans Christiaan Baaré, Kathrine Skak Madsen, Torben Ellegaard Lund, Leif Østergaard, Anders Juul, Troels Wesenberg Kjær, Carsten Hjorthøj, Hartwig Roman Siebner, Ole Mors, Merete Nordentoft

**Affiliations:** ^1^Child and Adolescent Mental Health Centre, Copenhagen University Hospital, Mental Health Services, Capital Region Psychiatry, Copenhagen, Denmark; ^2^Faculty of Health and Medical Sciences, Institute for Clinical Medicine, University of Copenhagen, Copenhagen, Denmark; ^3^The Lundbeck Foundation Initiative for Integrative Psychiatric Research (iPSYCH), Aarhus, Denmark; ^4^Research Unit CORE, Mental Health Centre, Copenhagen University Hospital, Mental Health Services, Capital Region Psychiatry, Copenhagen, Denmark; ^5^Department of Clinical Medicine, Faculty of Health and Medical Services, Aarhus University, Aarhus, Denmark; ^6^The Psychosis Research Unit, Aarhus University Hospital, Aarhus, Denmark; ^7^Child and Adolescent Mental Health Center, Roskilde, Denmark; ^8^Center of Functionally Integrative Neuroscience, Aarhus University Hospital, Aarhus, Denmark; ^9^Danish Research Centre for Magnetic Resonance, Centre for Functional and Diagnostic Imaging and Research, Copenhagen University Hospital Amager and Hvidovre, Copenhagen, Denmark; ^10^Department of Radiography, Department of Technology, University College Copenhagen, Copenhagen, Denmark; ^11^Department of Growth and Reproduction, Rigshospitalet, Section 5064, University of Copenhagen, Copenhagen, Denmark; ^12^Department of Neurology, Zealand University Hospital, Roskilde, Denmark; ^13^Department of Public Health, Section of Epidemiology University of Copenhagen, Copenhagen, Denmark; ^14^Department of Neurology, Hospital Bispebjerg, Copenhagen University, Copenhagen, Denmark

**Keywords:** familial high risk, schizophrenia, bipolar disorder, adolescent mental health, developmental trajectories

## Abstract

**Background:**

Children born to parents with severe mental illness have gained more attention during the last decades because of increasing evidence documenting that these children constitute a population with an increased risk of developing mental illness and other negative life outcomes. Because of high-quality research with cohorts of offspring with familial risk and increased knowledge about gene–environment interactions, early interventions and preventive strategies are now being developed all over the world. Adolescence is a period characterized by massive changes, both in terms of physical, neurologic, psychological, social, and behavioral aspects. It is also the period of life with the highest risk of experiencing onset of a mental disorder. Therefore, investigating the impact of various risk and resilience factors in adolescence is important.

**Methods:**

The Danish High-Risk and Resilience Study started data collection in 2012, where 522 7-year-old children were enrolled in the first wave of the study, the VIA 7 study. The cohort was identified through Danish registers based on diagnoses of the parents. A total of 202 children had a parent diagnosed with schizophrenia, 120 children had a parent diagnosed with bipolar disorder, and 200 children had parents without these diagnoses. At age 11 years, all children were assessed for the second time in the VIA 11 study, with a follow-up retention rate of 89%. A comprehensive assessment battery covering domains of psychopathology, neurocognition, social cognition and behavior, motor development and physical health, genetic analyses, attachment, stress, parental functioning, and home environment was carried out at each wave. Magnetic resonance imaging scans of the brain and electroencephalograms were included from age 11 years. This study protocol describes the third wave of assessment, the VIA 15 study, participants being 15 years of age and the full, 3-day-long assessment battery this time including also risk behavior, magnetoencephalography, sleep, and a white noise paradigm. Data collection started on May 1, 2021.

**Discussion:**

We will discuss the importance of longitudinal studies and cross-sectional data collection and how studies like this may inform us about unmet needs and windows of opportunity for future preventive interventions, early illness identification, and treatment in the future.

## Introduction

Parental mental illness is known to affect children in many ways, including risk of negative influence on upbringing circumstances, home environment, neurodevelopment, and increased risk of developing mental problems and disorders. This fact has been documented in several studies (1, 2), but less is known about how mental illness affects offspring during the adolescent years. As adolescence is a period characterized by significant changes in brain structure, connectivity, and functioning, as well as changes in physical appearance, hormonal status, and psychological and social constitution (3, 4), it is a period of life with dramatic development and changes. Adolescence is the time where the young person is searching for individuation and autonomy, while having a strong focus on peer relationships and at the same time start to separate from home and especially from the parents. It is concurrently the period with the highest incidence rates for mental disorders (3) and risk behavior (5). From a developmental perspective, it is a period in life that is highly not only formative but also challenging to study because of the complex interplay of biological (e.g., genetics, hormonal status, neuroplasticity) and social, environmental, and psychological (e.g., education, peers, sexual debut) risk factors.

### Brain Development

While early childhood includes the first and very sensitive periods for development of the sensory and motor systems (6), adolescence constitutes a second, but also very sensitive period for further development of the social, emotional, and higher cognitive domains (7). The networks that serve and constitute these brain functions are undergoing neuroplastic changes based on the experiences of the individual. The adolescent brain development can be characterized as a continuous maturation of cognitive functions mediated by higher associative cortices such as the prefrontal cortex including working memory, planning, concept formation, inhibitory control, and emotion regulation (8). A thinning of the cortex within the prefrontal cortex (and many other brain regions) and an increase in white matter density and volume are taking place (9, 10). In parallel, the brain is undergoing regional heterogeneous maturational changes with primary sensory and motor areas maturing before high associative cortical regions. Apparent cortical thickness is continuously decreasing from ~4 years of age, and surface area is increasing until early adolescence, whereas cortical gray matter volume steadily decreases after a peak in early adolescence (11). Such changes are thought to reflect both synaptic pruning and cortical myelination (12). White matter volume continues to increase into adulthood, with specific white matter fiber tracts displaying heterogeneous maturation with frontal–temporal association tracts such as the cingulum and uncinate fasciculus maturing well into adulthood (13).

With this maturation of complex structures and underlying brain networks, reflecting a high level of plasticity and learning potential, comes a heightened vulnerability to disease, disorder, and risk exposures that can compromise functional and structural maturation. External influence may lead to an increased possibility that functional and structural maturation can become abnormal and psychopathology may emerge (14). Puberty plays a role in brain maturation. Its onset in each individual varies widely, and so does its contribution (5, 15).

Adolescent brain development is not linear as the brain gets more connected and specialized in some areas, whereas others are reduced through a pruning process (16). The changing dynamic between frontal/executive and limbic/arousal/reward regions strongly influences the behavior of the individual. The malleability of the developing brain represents a high level of plasticity and learning potential but at the same time also represents vulnerability to disease, disorder, and risk exposures (14). Human brain development and functioning are also highly dependent on precise epigenetic regulation, and aberrant changes are increasingly reported to be associated with mental disorders (17, 18). Thus, DNA methylation plays a pivotal role in regulation of neuronal development and functioning, and its levels can be modified by environmental factors. Moreover, the genetic background of an individual is also associated with epigenetic variability, and risk single-nucleotide polymorphisms for mental disorders are reported to alter DNA methylation.

Adolescence is also the time for social transition from childhood to adulthood (7). In this transition period, research shows that young people are much more orientated toward and interested in their peers and how they look and behave than in adults (19). The social context is larger and more unpredictable, which implies a risk for social isolation, bullying, or peer rejection; it can be hard to cope with for vulnerable individuals (20). These processes and changes involve the networks of social cognition including mentalization and emotion regulation, which are some of the latest developed areas in humans. Good emotion regulation and well-developed mentalization (i.e., ability to think about others' thoughts, intentions and preferences) are protective against misunderstanding or interpreting others' behavior as directed negatively toward one self and to help to adapt to a stressful social situation [e.g., a peer rejection (20)]. On the other hand, these processes may also be involved in risk-taking behavior like experiments with drugs and alcohol or deliberate self-harm behavior (21).

In summary, adolescence can be understood as a window of vulnerability due to the significant neural changes, the changes in social roles, the onset of puberty, the increased risk of substance abuse, and other kinds of risk behavior, which can explain why the adolescent is at an increased risk of developing depression, psychosis, and many other mental health problems (22). A thorough review of the current knowledge and evidence on adolescence, brain development, and psychopathology can be found in *Biological Psychiatry* (23), where this was the special theme for the full issue (https://doi.org/10.1016/j.biopsych.2020.06.012).

### Familial High-Risk Studies

Schizophrenia and bipolar disorder are among the most costly and debilitating disorders both in terms of personal suffering for those affected, for the children and other relatives, and for society (24). Identifying disease mechanisms and possibilities for prevention before onset of illness will therefore be extremely valuable. As schizophrenia and bipolar disorder are rare conditions in the general population, studies of enriched populations (like children with familial high risk) can be fruitful and provide insight into the early disease processes. Approximately 55% of the children born to parents with schizophrenia, bipolar disorder, and severe depression will develop some kind of mental illness themselves during early adult life (25). Thus, the offspring have both a higher risk of developing the same disorder as their parents, or another severe mental disorder.

Familial high-risk studies have been conducted for decades (1, 26, 27). Previous familial high-risk studies have reported neurointegrative problems, social impairments, poorer neurocognitive and neuromotor functions, and early symptomatology among offspring of parents with severe mental illness (1, 2, 26, 28–30). However, because of limitations in previous studies such as small sample sizes, poor representativeness and wide age ranges, high attrition rates, lack of specific measures that inform about the underlying neurobiological processes, and lack of longitudinal follow-up, it is not clear whether these abnormalities abate, prevail, or worsen (30) over time.

Most of the previous studies were mainly based on convenience samples and were thus not representative. They included only a single assessment during childhood, and participating children were in different age groups (1). Developmental trajectories require at least three assessments, and longitudinal clinical cohort studies are therefore very valuable, although time consuming and costly. Attrition/dropout rates can be high, too.

Former waves of the study presented in this article have documented that children born to parents with schizophrenia and bipolar disorder show signs of vulnerability in a range of domains. In the first wave, The Danish High-Risk and Resilience Study—VIA 7, we found that as a group children with familial risk for schizophrenia and to some extent also bipolar disorder at age 7 years were impaired in, for example, neurocognitive functioning (31–33), social functioning (34), motor functioning (35), and mental health (36–38), while also living in environments with poorer levels of stimulation and support (39). The Danish High-Risk and Resilience Study (40)—of which the third wave, the VIA 15 study, is presented here—has overcome the obstacles described above by recruiting a large sample through national registers, all in a narrow age range that has been maintained in all three waves. The longitudinal method allows inference about development in the repeated waves of cross-sectional examinations, ultimately following developmental pathways in the longitudinal design. Therefore, conducting regular follow-ups on the defined outcomes is crucial for the end results of the study.

Structural and functional brain changes are present in drug-naive adult patients with schizophrenia, and some of the strongest risk factors exert their influence already in the prenatal or perinatal period (41). Notably, structural and functional neuroimaging of a large group of familial high-risk children before and during puberty, using a longitudinal design, has never been carried out before (42). In a recent study of offspring with familial risk for schizophrenia and bipolar disorder, the analysis of structural and functional brain networks revealed prominent group differences in brain organization, comparing vulnerable groups within a broad age range, and a relatively small sample (43). Brain imaging before, during, and after puberty is lacking in order to study brain development during this crucial period in human life. No previous studies have performed follow-up magnetic resonance imaging (MRI) of the brain of a large group of adolescents with a familial predisposition for schizophrenia and bipolar disorder.

## Aims and Hypotheses

The overall aim of this third wave of The Danish High-Risk and Resilience Study—VIA 15 is to follow up on the already defined domains of development and function in order to describe developmental trajectories, which are of great importance for mental health. The domains are psychopathology, neurocognition, motor function, and somatic health including sleep, physical activity, social cognition and social functioning, structural brain development, functional brain development, and environmental risk assessment including family situation, childhood trauma, and risk behavior.

We aim to

(1) improve insight into early disease processes of schizophrenia and bipolar disorder including early symptom formation and psychopathology, impairments or delays of maturation in different domains of cognitive functioning including social cognition, and changes in brain structure and task-related brain activation;
(2) identify the influence of genetic, epigenetic, and environmental exposures by analyzing associations between outcomes, such as psychopathology, risk behavior, and social and cognitive functioning, and structural and functional brain readouts and exposures, such as polygenic risk scores for schizophrenia, major depressive disorder, and educational attainment, and direct and indirect measures of the emotional climate in the family;
(3) identify early modifiable risk and resilience factors, such as low levels of stimulation and support in the home, traumatic life events during childhood, conflicting relation parents, neurocognitive and social cognitive deficits, risk behavior, and early signs of psychopathology, leading to development of good prediction models; and
(4) communicate the very important knowledge gained in this project about a vulnerable and overlooked group of children and adolescents to professionals who work with this population.

## Methods

### Design

**Figure 1:**
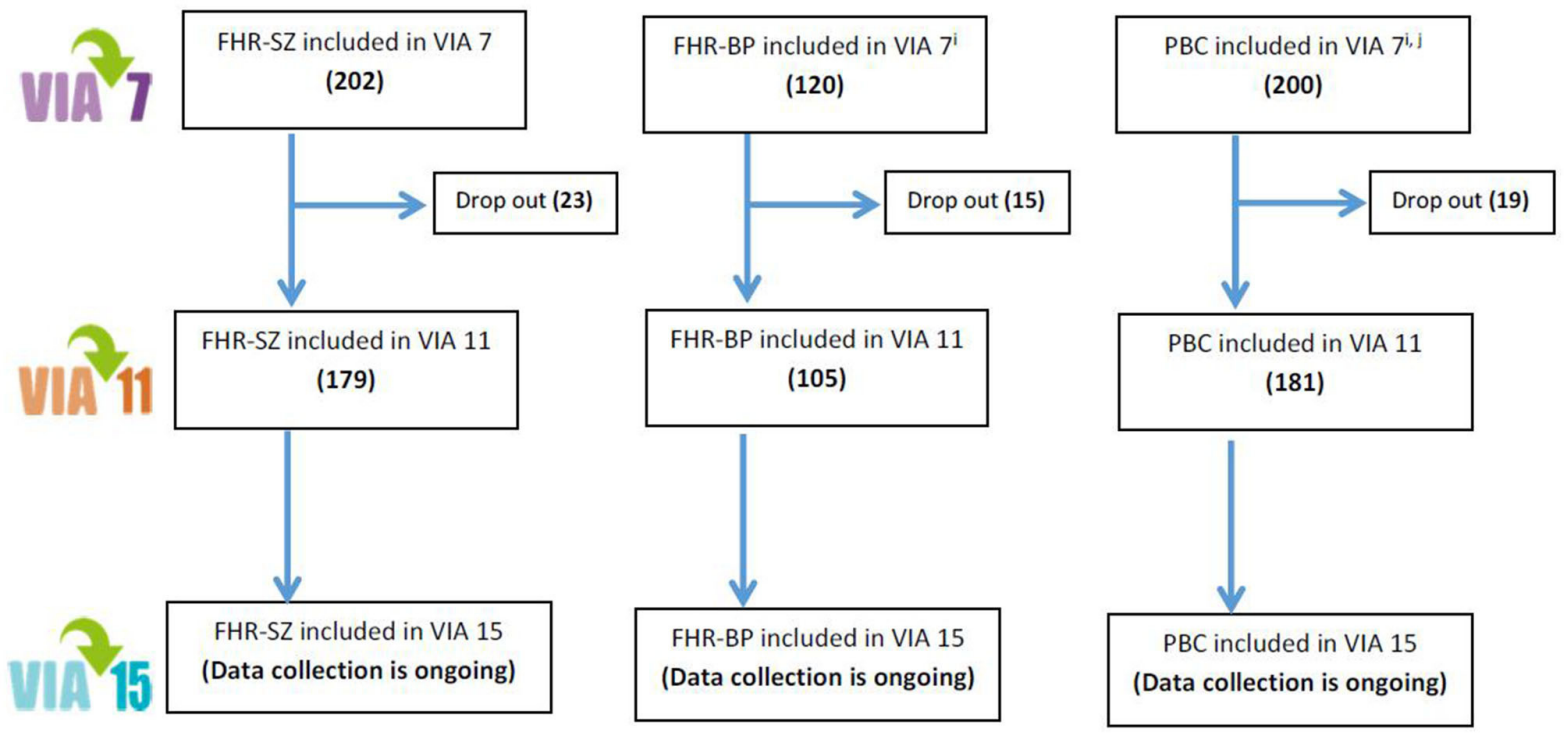
Flowchart of The Danish High-Risk and Resilience Study.

**Figure 2:**
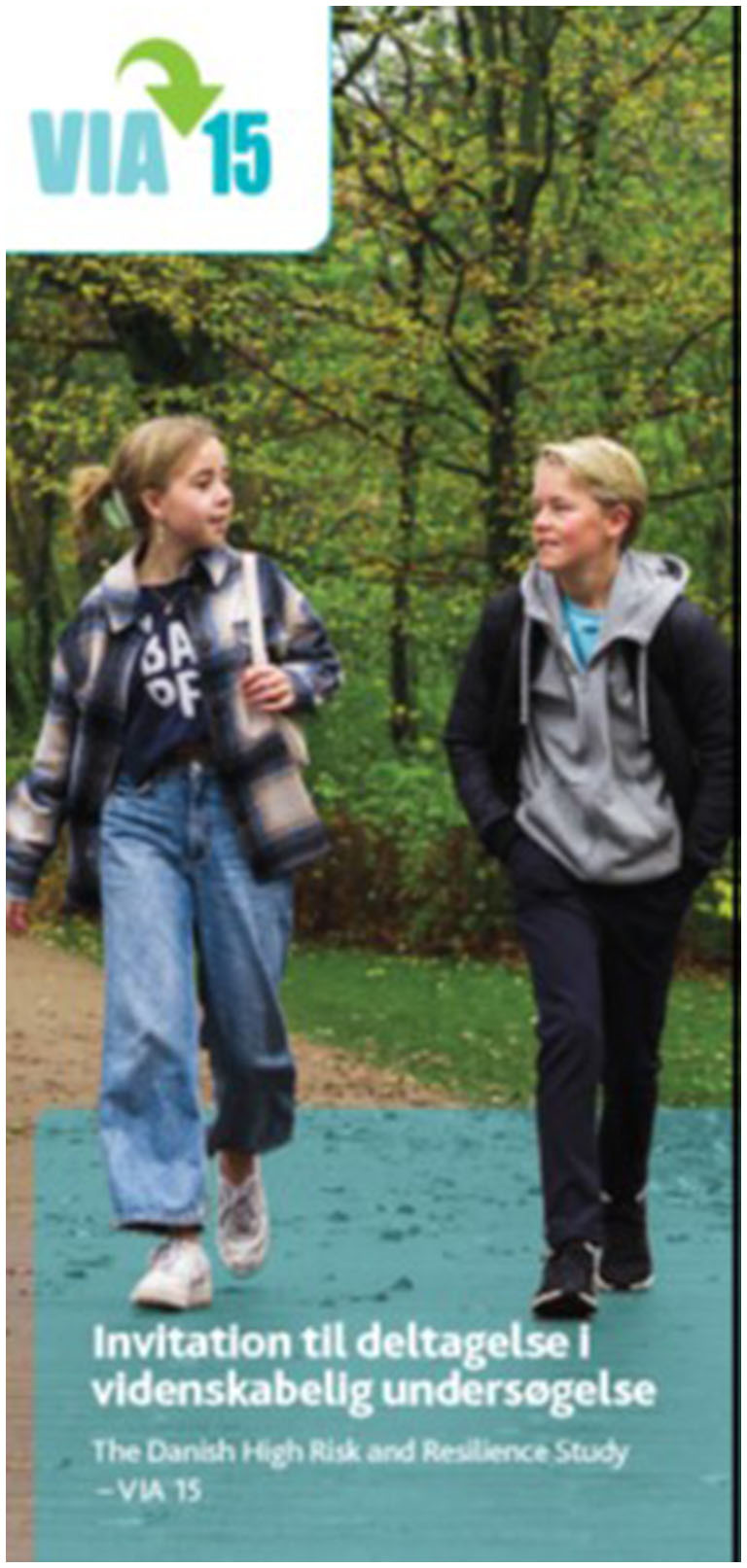
Image of the folder sent to all participating families in the VIA 15 study by mail.

The Danish High-Risk and Resilience Study is a representative nationwide longitudinal multi-informant cohort study consisting of 522 children born to parents with schizophrenia, bipolar disorder, or population-based controls. The participating families were recruited from Danish registers and investigated thoroughly during 2013–2015 when the children were 7 years old. This first assessment is referred to as the VIA 7 study (40). The second wave of assessments, the VIA 11 study (44), was carried out when the children were 11 years of age from 2017 to 2020 with an 89% retention rate. See Figure 1 for the flowchart and Figure 2 for image of recruitment folder sent to each family by mail.

The cohort consists of (a) 202 children with at least one parent diagnosed with schizophrenia spectrum psychosis (familial high risk of schizophrenia [FHR-SZ]); (b) 120 children with at least one parent diagnosed with bipolar disorder (familial high risk of bipolar disorder [FHR-BP]); (c) 200 children with neither of the parents treated in mental health services for the above diagnoses (population-based controls).

The control children were matched to FHR-SZ children on municipality, sex, and age. We included FHR-BP children as a nonmatched group; however, the group was comparable to the two other groups with respect to age and sex. The representative cohort is based on data from The Danish Civil Registration System (45) linked to the Danish Psychiatric Central Research Register (46). Analyses have shown that our cohort in many aspects is representative of the Danish population and have been described elsewhere (Falkenberg Krantz, submitted). Because of limitation of resources, we were able to include only 120 FHR-BP children.

### Earlier Assessments

In the VIA 7 study, saliva from the children and blood samples from the parents were used for genome-wide association analyses (GWASs, PsychChip). When the children were 7 and 11 years of age, the children and their parents were thoroughly examined with interviews, neurocognitive and social cognitive tests, questionnaires, home visits, and observations. In the VIA 11 study, MR scans and electroencephalographic (EEG) recordings were also performed. Assessments were supplemented with data from questionnaires sent to schoolteachers. Outcomes for the children were assessed thoroughly in the domains of neuromotor functioning, neurocognitive and social cognitive functioning, social functioning, and psychopathology at both ages. In addition, parents were interviewed about their mental health, and data on their neurocognitive functioning were collected. All assessors were kept blinded to whether the children were at familial high-risk or were population-based controls. Registration of unblinding in the former waves showed that assessors were unblinded in ~10% of all families.

The full assessment batteries in the VIA 7 and the VIA 11 study lasted ~3 days, and the vast majority of the families completed the whole battery. Parents were always offered feedback on their child's performance, and all participants received a gift card for their time taken, and practical obstacles such as transportation and catering were taken care of by the researchers. For families traveling longer distances, a hotel stay will be arranged for each family, like in the former waves. All families were informed at their previous visit that a follow-up at age 15 years was being planned.

### Assessment in the VIA 15 Study

The test battery in the VIA 15 study primarily focuses on the adolescent offspring, whereas only the primary caregiver's current level of daily functioning will be assessed with the Personal and Social Performance Scale [PSP Interview (47)]. Like in the former waves, a “primary caregiver” will be identified in each family (this may be a biological parent but could also be another adult), that is, a person, who is responsible for the adolescent's well-being on a daily/regular basis and preferably lives at the same address as the adolescent. This person may thus be different from the “primary caregiver,” who participated in the VIA 7 or the VIA 11 study. The primary caregiver will be asked to give information about the adolescent's mental health status and daily functioning both in interviews and from questionnaires.

The test battery for the adolescent will last ~3 days (5–6 h duration per day including breaks) and one night's sleep. Most tests and interviews will take place at the research clinic, unless the family for some reason needs the assessment to take place somewhere else, for example, in their home. However, measurement of sleep and assessment of the home environment will always take place in the home of the adolescent. All outcome measures are being examined with validated instruments, specifically developed and selected for this age group, sensitive to small changes, and suitable for later follow-up. Many variables will be measured for the third time, making analyses of trajectories possible. The battery consists of interviews, tests, observation, and questionnaires (Table 1). The battery is comprehensive, and some may find it exhausting, but individual needs (e.g., for breaks or shorter test days) are always taken into account to ensure a positive experience for the participants. As in the former waves, all adolescent assessors will be blinded to the familial risk status of each family.

**Table 1:** Assessment battery for the adolescents in The Danish High Risk and Resilience Study VIA 15, all domains.

**Domains**	**Outcomes**	**Instrument**	**Type of test**	**Approximate duration**	**In VIA 7**	**In VIA 11**
Neuromotor and physical measures	Motor function: manual dexterity, aiming & catching and balance	Movement Assessment Battery for Children-2 (Movement ABC-2)	Test	45 min	Yes	Yes
	Anthropometry	Measure of height, weight, waist	Observations in clinic	5 min	Yes	Yes
	Physical activity and sleep	SENS chip	Chip on thigh for 1 week	5 min + 1 week	No	Yes
	Polysomnography	Polysomnogram (PSG)	PSG monitor	Overnight	No	No
		Pittsburgh Sleep Quality Index (PSQI)	Questionnaire	5–10 min	No	No
	Motor screening	Motor Screening Test (MOT) from CANTAB	Computer test	5 min	Yes	Yes
	Smell identification	Brief Smell Identification Test (B-SIT)	Test	10 min	Yes	No
Neurocognition	Verbal Memory and visual memory	Word Selective Reminding from the Test of Memory and Learning–Second Edition TOMAL-2	Test	10 min	Yes	Yes
		Rey's Complex Figure Test (RCFT)	Test	8–10 min	Yes	Yes
	Attention	Rapid Visual Information Processing (RVP) from CANTAB)	Computer test	10–15 min	Yes	Yes
	Flexibility and processing speed	Trail Making Test 2-4 from Delis-Kaplan Executive Function System (D-KEFS)	Test	8 min	Yes	Yes
		Symbol Search and Coding test from Wechsler Intelligence Test for Children – Fourth Edition (WISC-IV)	Test	5 min	Yes	Yes
		Verbal Fluency 1-2 from Delis-Kaplan Executive Function System (D-KEFS)	Test	4 min	Yes	Yes
	Executive functions (planning and flexibility)	Stockings of Cambridge (SOC) and Intra-Extra Dimensional Set Shift (IED) from CANTAB	Computer test	20–30 min	Yes	Yes
		Verbal Fluency 3 from Delis-Kaplan Executive Function System (D-KEFS)	Test	2 min	Yes	Yes
	Executive functions (error monitoring)	Flanker Task	Test before and during fMRI		Yes	Yes
	Executive functions (visual and verbal working memory)	Spatial Working Memory (SWM) from CANTAB	Computer test	10-15 min	Yes	Yes
		Letter-number Sequencing and Arithmetic from Wechsler Intelligence Test for Children – Fourth Edition (WISC-IV)	Test	10–15 min	Yes	Yes
	Social cognition	Human Connectome Project Social Cognition Paradigm	Test during MRI	10 min	No	Yes
		The Animated Triangles Task	Test	12 min	Yes	Yes
		The Awareness of Social Inference Test – Part A2 (TASIT A2)	Test	10 min	No	No
	Intelligence	Reynolds Intellectual Screening (RIST)	Test	15 min	Yes	Yes
Psychopathology	Psychiatric symptoms	Kiddie Schedule for Affective Disorders and Schizophrenia (K-SADS-PL)	Interview	45–100 min	Yes	Yes
	Psychotic experiences	PE Hallucinations and PE Delusions	Interview inspired from the SOPS scale	1–10 min	Yes	Yes
	General functioning	Children's Global Assessment Scale (C-GAS)	Interview	20 min	Yes	Yes
	Speech illusions	White Noise Test	Test	20 min	No	No
	Risk factors for mental illness	Youth Experience Tracker Instrument (YETI)	Questionnaire	3–5 min	No	No
	Self-harm	Semi structured interview adapted to use in VIA 15 with items from Deliberate Self-Harm Inventory – Youth Version (DLSH-I) and iClinician-Administered Non-Suicidal Self-Injury Disorder Index (CANDI)	Interview	5–10 min	No	No
	Dimensional psychopathology	Youth Self Report for age 11-16 (YSR)	Questionnaire			
Social function and behavior	Behavior, affect, and test-taking-style	Tester's Observation Form (TOF)	Clinician rating	5–10 min	Yes	Yes
	Self-esteem	I think I am (Sådan er jeg”)	Questionnaire	5 min	Yes	Yes
	Bullying	Semi structured interview based on Olweus Bully/Victim Questionnaire	Interview	1–10 min	No	Yes
	Resilience	Child and Youth Resilience Measure (CYRM-12)	Questionnaire	1–3 min	No	Yes
	Social functioning	Strength and Difficulties Questionnaire (SDQ)	Questionnaire	3 min	No	Yes
	Risk behavior	Adapted questionnaire from 2019 National Risk Behavior Survey (USA)	Questionnaire	5–10 min	No	No
Environment and emotional climate	Perceived social support	Multidimensional Scale of Perceived Social Support (MSPSS)	Questionnaire	2 min	No	No
	Expressed emotions/ emotional family climate/familiar relations	Five Minute Speech Sample (FMSS)	Interview	7 min	No	No
		Family Assessment Device (FAD)	Questionnaire	2 min	No	No
	Social network and contact	Social contact questionnaires (from Lasgaard et al.)	Questionnaire	2 min	No	No
	Childhood trauma	Childhood Trauma Questionnaire, short form (CTQ-SF)	Questionnaire	3–5 min	No	No
Biological measures and physical health	Pubertal status	Tanner stages incl. menarche	Illustrations, test	3 min	No	Yes
		Hormone level	Blood sample		No	Yes
	Physical health	HbA1c. leucocytes, CRP	Blood sample		No	Yes
	Stress, biological measure	Hair test for long term level osf cortisol	Hair sample	5 min	Yes	Yes
	Bodily distress	Body Distress Symptoms checklist (BDS)	Questionnaire	2 min	No	No
	Health anxiety and somatization	Whiteley Index 6-R (Wi-6)	Questionnaire	1 min	No	No
Genetic and epigenetic analyses	Polygenic risk scores	Blood sample, saliva sample, dry blood spot and dry blood spots from Danish Neonatal Screening Biobank	Blood samples	5–10 min	Yes	Yes
	Inflammatory and infectious markers	Blood samples and dry blood spots	Blood sample	5–10 min	No	Yes
Brain scan	Brain structure and brain activity	Functional and Structural MRI and EEG	Brain scan at hospital	90 min	No	Yes
	Electrophysiology (only in Copenhagen)	The Copenhagen psychophysiological Test Battery: 40 Hz auditory steady state response Mismatch negativity Modified Eriksen Flanker task	Brain scan at hospital	120 min	No	Yes
	Magnetoencephalography (MEG, only Aarhus)	Paradigms: Roving auditory oddball + 40 Hz auditory steady state response	Brain scan at hospital	90 min	no	no

The assessors are highly skilled and educated psychologists, doctors, and research nurses who have been part of the preparation phase in the VIA 15 study and are trained and accredited in all tests and interviews. Weekly clinical conferences will be held in order to ensure uniformity between sites and testers, and a specialist in child and adolescent psychiatry (A.A.E.T.) will be present when psychiatric diagnoses are determined. For some instruments, interrater reliability will be measured [Vineland (48), Movement Assessment Battery for Children [ABC] (49)], whereas for others [Children's Global Assessment Scale [C-GAS] (50), PSP (47), psychotic experiences [PEs]], ratings will be made in consensus.

### Overview of Domains and Instruments in the via 15 Study

#### Adolescent Assessment

##### Neuromotor Function

Manual dexterity, ball skills, and balance are assessed with Movement ABC-2 (49), a clinical, gold-standard test for motor function that has also been used in the two former waves. To investigate manual dexterity, the participants will also be performing a circle-drawing task with their right and left hands on a pressure-sensitive digitizing tablet (WACOM Intuos4 large PTK-840; Wacom Technology Corporation, Vancouver, WA, USA) recording their writing trace from which the kinematics of the movements can be derived (e.g., movement velocity, frequency, and variability) (51).

##### Neurocognitive Function

Neurocognitive functions will be assessed with Rey's Complex Figure Test (52), Rapid Visual Information Processing [from Cambridge Neuropsychological Test Automated Battery [CANTAB] (53)], Verbal Fluency 1–3, and Trail Making Test conditions 2–4 A/B from the Delis-Kaplan Executive Function System (54), Symbol Search and Coding from the Wechsler Intelligence Test for Children—Fourth Edition [WISC-IV (55)], Stockings of Cambridge, Intra–Extra Dimensional Shift and Spatial Working Memory [from CANTAB (28)], Letter–Number Sequencing and Arithmetic [WISC-IV (55)] Word Selective Reminding from the Test of Memory and Learning—Second Edition (56), and Reynolds Intellectual Screening Test (57). Smell identification is measured with the Brief Smell Identification Test (58).

##### Social Cognition

Social cognition is measured by Animated Triangles (59, 60), consisting of short movie clips with two animated triangles moving around either in an intentional or arbitrary manner (note that the Animated Triangles Task measures theory of mind, a social cognitive domain), Emotion Recognition Task [from CANTAB (28), The Awareness of Social Inference Test—Part A2, and the Social Cognition paradigm from the Human Connectome Project (61) (performed during MRI).

##### Psychopathology

General psychopathology and PEs will be examined with the gold-standard diagnostic interview Kiddie Schedule for Affective Disorders and Schizophrenia [K-SADS-PL (62)]. This interview also includes a score based on a general assessment of the adolescent's daily functioning in the current month, the C-GAS (50). As before, we will include a specialized assessment of subthreshold psychotic-like experiences (PEs) inspired from the Scale of Prodromal Symptom Scale (63). Possible diagnoses and all PEs will be discussed at clinical conferences with a child and adolescent psychiatrist present. We used a modified version of the Attention Deficit/Hyperactivity Disorder Rating Scale [mADHD-RS (64)] to assess symptoms of attention-deficit/hyperactivity disorder and oppositional defiant disorder, rated both by the primary caregiver and the teacher. Affective liability will be measured using Children's Affective Liability Scale [CALS (65)]. We will also include Youth Experience Tracker Instrument [YETI (66)], a new brief self-report measure designed to facilitate early identification of risk for severe forms of mental illness, including major depressive disorder, bipolar disorder, and schizophrenia. By using the white noise paradigm (67), we will be able to investigate if a subgroup of children is more likely than the others to appraise an ambiguous situation as, for example, threatening. We will apply a Danish version of the white noise paradigm, which is a series of 75 very short sound clips with white noise. In two of three sound clips, short and neutral sentences are included in the sound of the white noise, 25 clearly audible and 25 barely audible, whereas the remaining 25 sound clips included only white noise. The respondents can select the following responses: 1 = “hearing positive voice,” 2 = “hearing negative voice,” 3 = “hearing neutral voice,” 4 = “no speech heard,” and 5 = “uncertain.”

Data from school will also be included via questionnaires sent to the schoolteachers if parents give permission (i.e., sign a consent form). Executive functioning including affective regulation and flexibility will be assessed with the questionnaire Behavior Rating Inventory of Executive Function [BRIEF (68)] from both the primary caregiver and the teacher. Autism spectrum traits are evaluated with Social Responsiveness Scale [SRS (69)] also completed by the caregiver and the teacher. Dimensional measures of psychopathology will be covered with Youth Self-report version of the Child Behavior Checklist [CBCL (70)] and also from the primary caregiver and the teacher. The adolescent will also be asked to complete the Strengths and Difficulties Questionnaire (71). The ratings of the clinical impression of the adolescent during the testing are reported with Tester's Observation Form (72). All the mentioned questionnaires have been used in the VIA 7 study and in the VIA 11 study as well.

##### Social Functioning, Self-Esteem, Deliberate Self-Harm, Risk-Taking Behavior, and Resilience

Adaptive social functioning of the adolescent is captured by parental interview using the Vineland-2 (48). Self-esteem is covered by the questionnaire “Sådan er jeg” (“This Is Me”), a questionnaire about self-esteem in school, in the family, and in a peer context (73).

Deliberate self-harm is a questionnaire made by our own research group in collaboration with specialists in the area. We collapsed items from two longer questionnaires, the Deliberate Self-harm Inventory—Youth Version (74) and Clinician-Administered Non-Suicidal Self-injury Disorder Index [CANDI (75)] and will be administered as a semistructured interview in the VIA 15 study.

Risk-taking behavior will be assessed with a modified and adapted questionnaire based on Youth Risk Behavior Surveillance System (76), whereas school performance, leisure activities, social relations, and use of social media are included in the anamnestic interview (i.e., interview about what has happened in the adolescent's life within the previous 4 years, since the VIA 11 study) made primarily with the primary caregiver as informant. Alcohol and drug use is also covered by interview, partly as part of the K-SADS-PL (diagnostic level of misuse) and in a specific, short interview suited for this specific age group. Level of stress will be captured from hair cortisol. Perceived social support will be assessed with a questionnaire, Multidimensional Scale of Perceived Social Support [MSPSS (77)].

Resilience is measured by a short version of the questionnaire Child Youth Resilience Measurement—Youth Version (78). Affective regulation is captured by the questionnaire CALS (65).

##### Environmental Factors

The family environment in terms of family functioning will be assessed by both the parent and the adolescent by using the questionnaire Family Assessment Device [FAD (79)], which was also in the VIA 11 study. The 5 Min Speech Sample [FMSS (80)] was used in the VIA 7 and the VIA 11 studies for the primary caregiver to talk about the child, but this time it will be administered with both the primary caregiver and the adolescent. Adverse life events including unwanted sexual experiences will also be assessed by a questionnaire, Childhood Trauma Questionnaire—Short Form (81, 82), and is also included in the anamnestic interview. Further, childhood trauma is measured directly from the adolescent and the primary caregiver in the K-SADS-PL (62) interview section about traumatic events and PTSD. Social network is captured by MSPSS (77).

##### Biological Measures and Physical Health

We will make a clinical evaluation of anthropometry of the adolescent (height, weight, and waist circumference) at the time when the adolescent visits the clinic. Further, three different biological samples will be acquired, including a small hair sample to measure the levels of the stress hormone cortisol, a blood sample that will provide data on the immune system, diabetes, and so on, and a saliva sample used for genetic and epigenetic analyses. Physical activity will be measured by a sensor in an easily wearable adhesive patch [SENS motion® (83)], which directly measures the amount and level of physical activity during a 1-week observation period. Retrospective report on menarche and growth will be obtained, and puberty status will be assessed from the four Tanner stages by asking the adolescents to estimate their current developmental state from a figure (84, 85). Sex hormones (i.e., testosterone and estradiol) will be measured from the blood sample. Bodily distress symptoms are covered by the questionnaire Body Distress Symptoms checklist (86), and screening for somatization and hypochondriasis is covered by Whiteley Index 6-R (87).

##### Neuroimaging

*Structural and Functional MRI and Magnetoencephalography/EEG*. We will repeat the anatomical and functional MRI (fMRI) of the whole brain at 3.0 T, which was carried out at age 11 years. MRI with harmonized scan parameters will be performed at Aarhus University, Center for Integrative Neuroscience (CFIN) and Hvidovre Hospital, Danish Research Center for Magnetic Resonance (DRCMR). We will acquire three-dimensional high-resolution MP2RAGE structural scans and diffusion-weighted MRI to derive, respectively, measures of brain structure, including global and regional cortical thickness, area, volume, and gyrification; subcortical brain structure (and microstructure); and myelin sensitive brain tissue maps and microstructural properties of gray and white matter brain tissue (e.g., fractional anisotropy, mean diffusivity), as well as measures of structural connectivity by means of, for example, tractography and structural covariance. Task-related functional brain activity and connectivity will be assessed while participants perform well-established paradigms as in the VIA 11 study, that is, Eriksen Flanker Task (88) and the Social Cognition Task from the Human Connectome Project, that is, Animated Triangles Test (59, 60), which, respectively, probe executive cognitive control (89) (i.e., distractor resistance during fast response choices cued by directional cues) and social cognition (i.e., inferring the intentionality of moving objects^1^). In addition, and new to the VIA 15 study, we have included a reward paradigm. In the reward paradigm, participants start out with 100 DKK and are then repeatedly presented with two different stimuli in random order. Each stimulus presentation is accompanied with varying outcomes adding or subtracting to their current wealth. Participants thus can learn about the reward probability distributions of the two stimuli. The aim is to investigate whether the dopaminergic reward system represents the entire reward probability distribution, as recently suggested by an experiment in mice (90), and whether this neural distribution is changed in the high-risk groups. We have chosen these tasks because task-related networks are hypothesized to be implicated in the pathophysiology of neurodevelopmental disorders. Functional profiling of these brain systems will enable us to infer specific network properties and dynamics that contribute to disease formation or resilience.

*EEG (DRCMR only)*. We will repeat the EEG assessments performed in the VIA 11 study. Specifically, an auditory oddball paradigm to measure Mismatch negativity (91) and an auditory paradigm (using 40-Hz click trains) to measure steady-state oscillations (92) will be used. In addition, we will repeat the Eriksen Flanker task that is both performed during the fMRI and EEG.

By combining fMRI and EEG data (although not acquired concurrently), we will be able to get a deeper understanding of lower-order processing as well as the interaction of specific brain regions during the emerging of psychopathology, on the one hand, and cognitive control, on the other hand, during this age period.

*Magnetoencephalography (CFIN only)*. We will perform magnetoencephalographic (MEG) recordings of all participants assessed at the Aarhus study site, expecting a total sample size of 175–200. We will collect MEG data using the ELEKTA Neuromag TRIUX MEG system with 204 planar gradiometers and 102 magnetometers. Like EEG, MEG measures brain activity with high temporal resolution; however, MEG can achieve slightly higher spatial resolution compared with EEG. As for the EEG recordings, we will apply two auditory paradigms: the roving auditory oddball paradigm (to elicit mismatch negativity) and the 40-Hz auditory steady-state response, to investigate evoked and induced responses, respectively. Both paradigms are well-replicated in patients with both schizophrenia and bipolar disorder, with medium–large effect sizes, compared with healthy controls. Our MEG data can subsequently be combined with T1-weighted structural images from MRI scans for source localization. We will investigate effective connectivity within and between brain regions using dynamic causal modeling, which will allow us not only to investigate the clinical usefulness of two putative biomarkers for schizophrenia and bipolar disorder, but also to investigate the pathophysiological trajectory leading from a familial high-risk state to manifest illness.

##### Polysomnography

Polysomnography (PSG) is a noninvasive EEG-based method, considered the gold standard of sleep analysis, and widely applied both in clinical practice and for research purposes (93). We will examine the sleep pattern and sleep stage architecture of participants with PSG. For PSG recordings, we will apply a portable recording device, the Somnomedics Somno HD with the 32-channel Somnomedics EEG+ headbox attached to capture EEG signals from the scalp, electrocardiographic signal from the chest, electromyographic signals from the chin and thigh and electro-oculographic signals from the outer lateral canthus left and right sides. Electrodes will be placed according to the American Academy of Sleep Medicine guidelines for extended EEG montage. Trained personnel will fit the PSG equipment on location in each participant's home. Participants will wear the PSG equipment for one night at home, sleeping as normal. Next morning, after the recording, participants remove the equipment and store it for collection by our staff. Following the PSG recording, participants must complete the Pittsburgh Sleep Quality Index (94). Except for potential mild discomfort from sleeping with the equipment, there are no known adverse effects or complications to the method.

Data will be analyzed in order to score the expression of the various sleep stages based on the complete recording period, to produce a hypnogram for each participant. The occurrence of individual sleep spindles (95) and K-Complex' (96) in the EEG recording will be marked for each participant as well.

##### Genetic and Epigenetic Analyses

DNA samples were obtained from a subset of the VIA study sample, which included both parents and children. These were genotyped on the Illumina PsychChip v1-1_15073391_C. The genetic data were subject to quality control measures adapted for a family-based sample, as outlined in our previous papers (97, 98). Genetic analyses include family-based GWASs and analyses for the detection of parent-of-origin effects as well as generation of polygenic risk scores for use in downstream studies either directly or to account for genetic predisposition to an array of traits, including psychiatric disorders (e.g., schizophrenia) and physiological traits (e.g., body mass index [BMI]).

The VIA 7-11-15 studies have the unique opportunity to study neonatal epigenetic signatures from birth through childhood and adolescence toward development of mental disorders diagnosed later in life and integrate them with genetic and environmental data. We will additionally assay DNA methylation for all 522 children in peripheral samples collected at birth from dried and saved bloodspots (phenylketonuria test made at birth and stored for all children in Denmark) and at all three follow-up visits (the VIA 7 study, the VIA 11 study, and the VIA 15 study) to provide longitudinal assessment of epigenetic changes from birth and during child–adolescent development. Genome-wide DNA methylation will be assayed with the use of Infinium Methylation EPIC BeadChip (tagging 850,000 sites across the genome).

This epigenetic data will be subjected to stringent quality control and data processing using well-established Bioconductor packages (99–101). In order to account for cellular heterogeneity and reduce the confounding in the sample, we will predict blood cell proportions from the epigenetic data and further adjust for these measures in our association models (102). We will perform cross-sectional epigenome-wide association analyses to identify epigenetic markers of brain structure and activation, as well as social cognition, language, olfactory function, measures of hormones, and immune function. We will also investigate interaction scenarios between DNA methylation, genetics, and environmental exposures with measures of brain structure and functioning as outcome.

### Primary Caregiver Assessment

The primary caregiver is the actual caregiver and defined as an adult who knows the adolescent very well, lives with the adolescent, or has daily contact with and is caring for the young person and who can thus provide reliable information. The primary caregiver will be asked to participate in an anamnestic semistructured interview concerning the previous 4 years (since the assessment at age 11 years, the VIA 11 study) about development, school performance, and daily behavior of the adolescent. The primary caregiver will also be asked to provide information about the adolescent's mental health status through the K-SADS-PL interview (62) and from a series of questionnaires (Table 2). Further, the primary caregiver will be asked to give a short speech sample about the adolescent, the FMSS (80). The primary caregiver will be asked about his/her daily functioning during the previous month by the interview Personal and Social Provision Scale [PSP (47)], and the adult will be asked to fill in a questionnaire about the family functioning by the FAD (79) (Table 2).

**Table 2:** Assessment battery for the primary caregiver in The Danish High Risk and Resilience Study—VIA 15.

**Domains**	**Instrument**	**Type of test**	**Duration**	**In VIA 7**	**In VIA 11**
Family relations, education, stressors, health and social life	Anamnesis	Interview	30–40 min	Yes	Yes
Mental health status in adolescent	Kiddie Schedule for Affective Disorders and Schizophrenia (K-SADS-PL)	Interview	45–90 min	Yes	Yes
Attention/hyperactivity	ADHD-Rating Scale	Questionnaire	5–10 min	Yes	Yes
Executive functions	Behavior Rating Inventory of Executive Function (BRIEF)	Questionnaire	10 min	Yes	Yes
Autism spectrum traits	Social Responsiveness Scale (SRS-2)	Questionnaire	10 min	No	Yes
Affect regulation	The Children's Affective liability Scale (CALS)	Questionnaire	2–5 min	No	No
Social development	Vineland Adaptive Behavior Scales – II	Interview	20 min	Yes	Yes
Daily functioning	Personal and Social Performance Scale (PSP)	Interview	10 min	Yes	Yes
Family Functioning	Family Assessment Device (FAD)	Questionnaire	3 min	No	Yes
Environment and emotional climate	Five minutes Speech Sample (FMSS)	Interview	7 min	Yes	Yes
Behavior	Child Behavior Checklist (CBCL)	Questionnaire	10 min	Yes	Yes
Genetic and epigenetic analyses	Saliva sample	Saliva sample	5 min	Yes	Yes

### Teacher as Informant

If the parents give permission and the adolescent accepts it, a series of five questionnaires will be sent to the school teacher to ensure information from school: the SRS (69) the mADHD-RS (64), Teacher's Reports Form [similar to CBCL (70)], and BRIEF (68). See also Table 3.

**Table 3:** Assessment battery for the adolescents' teachers in The Danish High Risk and Resilience Study VIA 15.

**Domains**	**Instrument**	**Type of test**	**Duration**	**In VIA 7**	**In VIA 11**
Psychosocial functioning and behavior	Teachers Rating Form (TRF)	Questionnaire	10 min	Yes	Yes
Attention/Hyperactivity	ADHD-Rating Scale	Questionnaire	5 min	Yes	Yes
Affect regulation/flexibility	Behavior Rating Inventory of Executive Function (BRIEF)	Questionnaire	5 min	Yes	Yes
Autism spectrum traits	Social Responsiveness Scale (SRS-2)	Questionnaire	5–10 min	Yes	Yes
Communication and social interaction	The Children's Communication Checklist CCC-2	Questionnaire	10 min	Yes	Yes

#### Practical Issues

The dropout rate between the first and the second wave, the VIA 7 and the VIA 11 study was only 11%, and we believe that this has to do with our aim and great effort to meet each family with a friendly and flexible approach when arranging their participation. Therefore, as before, testing can be conducted over several days and take place at time points and places that suit the adolescents' needs and the families' specific preferences. If there are any special tests, interviews, or questionnaires that the informants for some reason do not want to take part in, this is always respected and will not lead to exclusion from the study. Transportation and catering are arranged in collaboration with the family. All participants will receive gift cards for their time taken, and travel reimbursement is offered.

Both the adolescent and the primary caregiver will be offered a verbal feedback with the conclusions from the assessments completed. Participation in the study does not include any interventions or treatment. In case of obvious needs for psychiatric treatment, or medical or psychological assistance, the adolescent (and the parents if the adolescent allows it) will be guided in how to find relevant assistance or help. In cases where referral to secondary mental health service system (i.e., hospital treatment) is urgent, we will make the referral immediately. Health professionals including researchers are obliged to make referrals to the Child Protection Services in the municipalities, when needed (in some cases without consent if the problems revealed are very serious). When milder problems arise during the assessment, the researchers will give the adolescent a list of public and nongovernmental organizations (NGOs) and institutions, which can be contacted without referral, including telephone counseling, chat forums, and open-door services.

### Funding

The VIA 15 study has received financial support from The Lundbeck Foundation: 20 million DKK (~2.6 million euros), The Novo Foundation: 10 million DKK, Mental Health Services, and Capital Region of Denmark: 10 million DKK (~1.3 million euros). Further financial support is currently being sought to cover extra costs including delays caused by COVID-19 and other unforeseen events.

### Ethics and Data Protection

A minor proportion of the families, who participated in the VIA 7-study, declined to participate in the VIA 11 study for various reasons (11% of all). This information is carefully registered in our cohort files. All data from the VIA 7 study are stored at Statistics Denmark and linked to register-based information about use of mental and somatic health services for parents and children [National Patient Register (46, 103)], parental education, and source and level of income (104). Data from the VIA 11 study and the VIA 15 study will also be stored at Statistics Denmark. The study protocol was approved by the ethical committee in March 2021 (Journal-nr.: H-20067908), and all guidelines and regulations for data security and data protection are being followed carefully. Data collection started on May 1, 2021, and all data are collected and stored in REDCap^2^ (105).

### Statistics

The analyses from the VIA 7 study have shown that the sample is large enough to show group differences of 0.25 *Z* scores and larger in tests of neurocognition and social cognition. The size of the sample allows for analyses of mediation via home environment or other environmental exposures from the VIA 7 and the VIA 11 studies and for latent class analyses of trajectories.

Differences between the three groups will be analyzed with multivariate and univariate analyses of variance or χ^2^ test as appropriate. Between-group differences of diagnoses will be evaluated using logistic regression adjusting for the adolescent's sex. Multiple imputations will be applied with 20 imputations using a multivariate normal distribution. Multiple imputations will be followed by a standardizing of continuous data into *z* scores, using the control mean as reference. Mixed models, Cox regression, and latent class growth analysis will be applied in the longitudinal data analyses.

## Results

Results will be presented within the context of both cross-sectional and longitudinal analyses, that is, comparing the results from the first assessments at ages 7 and 11 years, this time giving us the opportunity to present developmental trajectories with three time points. Results will be reported in all domains that have been included from the outset (neurocognition, psychopathology, social behavior, and social cognition and daily functioning, motor function, and family/home environment). Follow-up on the domains introduced in the VIA 11 study (i.e., MR scans, EEG recordings, SENS motion data, and blood sample data) will be of special interest. In addition, for many of the domains covered partly by questionnaires, we can at this time create trajectories and compare the three groups with the exact same instrument. Results concerning the actual indicators of subthreshold psychopathology and symptoms that meet the diagnostic criteria will be analyzed into mental health status at ages 7 and 11 years. This time, we will be able to include data on deliberate self-harm, risk-taking behavior including alcohol and drug use, reports of current or previous experiences of social exclusion, or bullying and physical health (immune system status, BMI, sleep, etc.). Finally, we will be able to investigate how differences and/or changes in structural and functional brain readouts are related to differences and/or changes in clinical and behavioral measures and how these are modulated and/or mediated by biological and environmental factors.

For all domains, we have strived to use instruments that can be used for a wide age span. We therefore have a huge amount of data with similar methods, and analyses will take into account to what extent these children's deficits or advantages measured at ages 7 and 11 years remain stable, deteriorate, or diminish over time.

## Discussion

In this article, we have described the outline for the third wave of The Danish High-Risk and Resilience Study—VIA 15, a follow-up study on 522 children born in Denmark, most of them with a familial predisposition for schizophrenia or bipolar disorder. We aim to follow up on all the central domains that have already been thoroughly investigated at ages 7 and 11 years, and thus, we will be able to demonstrate trajectories for both good and poor outcomes and at-risk states in adolescence. The overall purpose is to contribute to the existing knowledge about etiology and development of mental illness and to propose optimal time points and domains or specific profiles relevant and especially targeted preventive strategies and early interventions for offspring with familial risk for severe mental illness.

There are other research groups around the world, who are also assessing children with familial risk for both schizophrenia and bipolar disorder, and some also include children born to parents with moderate to severe depression (106). Those who are closest to our study are the Bipolar and Schizophrenia Young Offspring Study in Spain (107) and the Families Overcoming Risks and Building Opportunities for Well-being Study in Canada (108). The latter is also testing different models of interventions, while investigating antecedents, symptom development, and behavior while in the same study testing different models of interventions. These and other familial high-risk studies have confirmed what earlier high-risk studies revealed, namely, that the increased risk for the offspring to be ill is not specific for the illness of the parent, but rather is seen as a generally increased risk for developing any mental disorder (25). Further, research has shown that a developmental perspective is needed when trying to disentangle, understand, and interpret the importance of unspecific and early mental health problems and subthreshold symptoms in terms of seeing these early signs as markers of emerging psychiatric disorders (109, 110).

The Danish High-Risk and Resilience Study—VIA 15 also implies some challenges that we are completely aware of. Of utmost importance is the willingness of the families to participate again. From the former waves, we already learned that practical issues and logistics such as arranging transportation and remembering exact time and meeting point can be troublesome especially for the families, who struggle with ongoing or acute episode of mental illness. In the VIA 11 study, we saw that some of the children already at that age had developed various mental problems that could make further participation difficult for them (111). A 15-year-old teenager will have more influence on the decision, and if he/she is reluctant, the primary caregivers may not want to force/put pressure on him/her. On the other hand, at age 15 years, the adolescents are familiar with the study from the former waves, and many of them expressed that they liked being part of it. Most of them will still be in elementary school, and not in high school, which may also make participation a bit easier to find time for.

For domains such as psychopathology, risk behavior, and social relations, we know that being 15 years of age implies some very specific behavioral patterns and social processes that we need to be aware about and well-educated to capture and document. For example, we expect that adolescents at age 15 years will present with mental health issues that include symptoms from many different diagnostic entities and when an exact diagnosis can be hard to determine (112). This age group often present with many mild to moderate transdiagnostic symptoms pointing in different directions (113). For example, mood swings, deliberate self-harm, isolation, and some irregular alcohol use can be both normal teenager problems and signs of underlying mental disorder. Therefore, this must be scrutinized in order to avoid overdiagnosing or underdiagnosing. Some of the young people may also describe some more subjective changes in sensory functions such as heightened perceptions of light or sound and self-disturbances that may be early warnings of later psychotic illness, which to some degree is covered by the questionnaire YETI (66) but not in the K-SADS-PL (62).

If the current situation is evaluated to be acutely unsafe and the adolescent's health situation is at risk, we will offer a statutory referral to the municipality's Child Protection Services or to the Center for Child and Adolescent Mental Health Services, depending on the type of problem presented. For less severe or acute cases (e.g., few occasions of deliberate self-harm that happened some months ago), we will hand out a list of low threshold, that is, easy to access and open-door services and organizations, which can be contacted for all kinds of unspecific problems with school, parents, friends, and peers, to get advice and support (e.g., headspace and other NGOs, general practitioners, municipality's open office, telephone counseling, and chat forums). We will also provide feedback on the test results to both the adolescent and the parents if they wish.

For some of the adolescents with familial high risk for mental disorder, the transition to adulthood can be troublesome for other reasons than those shared by everyone. Being a relative to a person with a severe mental illness can seriously impact daily life functioning and behavior, both for the other parent, often identified as the “well parent,” and for the children. The process of finding one's own identity, being more independent, expressing oppositional viewpoints, and separating from the home and the family structure is a natural process in this phase of life. But in families with parental mental illness, these processes can be much more difficult, if the adolescent at the same time has a huge responsibility for practical tasks in the family or for the emotional and psychological well-being of the parent. Many adolescents worry about what will happen to their ill parent, if they stay out long or even leave home 1 day, and some have a very close relationship to the parent, which makes it difficult to be an individual without thinking about the parent's needs (114).

### Potential Clinical Implications

Prevention and early intervention are important and possible and are being developed and tested in many areas of psychiatry (115). Children born to parents with severe mental illness have been overlooked and “fallen between chairs,” but longitudinal studies such as this can help change that. Early intervention programs can be developed and tested in accordance with knowledge about the children's developmental trajectories and early signs of mental illness with specific focus on various outcomes. A recent review of intervention studies targeting children with familial risk for mental disorder documented that it is possible to influence the risk profiles of the individuals by rather simple, general, or short interventions (116). Risk of mental illness was reduced as were both internalizing and externalizing symptoms. Interventions varied a lot but were primarily cognitive behavioral therapy, parental training, and psychoeducation. Some of the factors that children born to parents with mental illnesses live with are shared with children in families, where a parent has a serious somatic disorder, such as worrying about the parent, being a “young carer.” Other similarities include having a hospitalized parent, whereas other factors are more specific (change of the personality, emotions, and behavior and high levels of stigma). When children are relatives, the parent's illness poses a risk on their environment, which could be further included in treatment and prevention strategies by providing information and knowledge to the children about the parent's situation (117).

One of our long-term goals of the comprehensive study is to be able to—on the basis of the results from the three measurements—detect the most vulnerable individuals by assessing their profiles at an early time point and use this knowledge to inform intervention studies and develop specialized interventions that are directed against the specific problems or symptoms that they display. However, most of the knowledge about preventive interventions for children and adolescents emphasize the importance of also including parents and other important adults around the child/adolescent at risk to have a more holistic approach. Also, school, social environment, access to leisure time activities, and local communities have a potentially important role in providing options for resilience and self-esteem (115).

## Conclusion

Longitudinal studies are time- and resource-consuming but have a major potential for highlighting developmental processes for individuals with familial risk of severe mental illness such as schizophrenia and bipolar disorder. The large and unique cohort of 522 individuals in The Danish High-Risk and Resilience Study has already provided striking results in terms of higher rates of early markers of vulnerability, developmental delays, and clinical problems compared with population-based controls. The cohort is now being followed up for the third time to inform preventive strategies and early interventions in the future.

## Ethics Statement

The studies involving human participants were reviewed and approved by the Ethics Committee of the Capital Region of Denmark. Written informed consent was obtained from the individuals and minors' legal guardian/next of kin for the publication of any potentially identifiable images or data included in this article.

## Author Contributions

AT and MNo wrote the manuscript. All authors contributed to development of study design and preparation of the manuscript, and have commented and approved the submitted version.

## Funding

This work was funded by Lundbeckfonden—R-277-2018-594, MEG part: Lundbeckfonden—R322-2019-2711, Novo Nordisk Fonden—NNF20OC0060468, and Region Hovedstadens Psykiatris Forskningspulje.

## Conflict of Interest

The authors declare that the research was conducted in the absence of any commercial or financial relationships that could be construed as a potential conflict of interest.

## Publisher's Note

All claims expressed in this article are solely those of the authors and do not necessarily represent those of their affiliated organizations, or those of the publisher, the editors and the reviewers. Any product that may be evaluated in this article, or claim that may be made by its manufacturer, is not guaranteed or endorsed by the publisher.

## Footnotes

^1^Note that The Animated Triangles Task and the social cognition paradigm from The Human Connectome Project are not exactly the same. The latter both contain triangles, squares, and circles. However, the premise is the same (the figures are moving around either in an intentionally or random manner).

^2^Study data were collected and managed using REDCap electronic data capture tools hosted at Mental Health Services, Capital Region of Denmark.1 REDCap (Research Electronic Data Capture) is a secure, web-based application designed to support data capture for research studies, providing (1) an intuitive interface for validated data entry, (2) audit trails for tracking data manipulation and export procedures, (3) automated export procedures for seamless data downloads to common statistical packages, and (4) procedures for importing data from external sources.
